# Epistemic Humility in the Age of Assisted Dying

**DOI:** 10.1002/hast.4960

**Published:** 2025-04-17

**Authors:** Sean Riley

**Keywords:** medical assistance in dying, MAID, end‐of‐life policy, epistemic humility, research design, data validity, research ethics

## Abstract

*The current debate on medical assistance‐in‐dying (MAID) fails to acknowledge the limitations of empirical data and the influence that cognitive biases exert in interpreting evidence and formulating arguments. This paper examines the evidentiary foundations of the MAID debates by conducting a critical analysis of the methodological approaches to research on MAID. The paper advocates for epistemic humility in this debate, including the acknowledgment of the fallibility of MAID research, the incompleteness of understanding surrounding MAID, and the limited usefulness of empirical facts in determining ethical judgments. These factors cast doubts over the role data can play in shaping MAID discourse. Developing a well‐balanced MAID policy necessitates an innovative research framework that not only prioritizes methodological rigor and data integrity but also integrates ethical deliberation with empirical research through a commitment to epistemic humility*.

## Essays

The past decade has seen heated academic and political debate over medical assistance in dying (MAID). Many jurisdictions, across four continents, have legalized the practice for the first time, including Austria, Canada, Colombia, Ecuador, New Zealand, Portugal, Spain, and several states in Australia and the United States.^1^ Further, several jurisdictions have liberalized their existing MAID laws to reduce administrative burdens and expand patient access.^2^ The question of whether we should allow MAID to be practiced legally is transforming, now that MAID is legal, into a question of how we should practice and regulate it. In 2021, Canada's Bill C‐7 eliminated waiting times, witness requirements, and the prohibition of mental illness as a qualifying medical condition (though the provision for people with mental illness is yet to be implemented).^3^ In 2023, the Netherlands expanded access to MAID for all terminally ill children, now permitting it for those younger than twelve years old.^4^ A similar expansion was passed in Belgium in 2014.^5^ In 2024, a bill in the California senate aimed to remove the terminal illness requirement common in U.S. MAID policies and to shift to the “grievous and irremediable” language used in other parts of the world, although the bill has yet to leave committee.^6^

Each of these examples illustrates how an evolving understanding of how MAID operates has affected the views of the public, policy‐makers, advocates, and opponents alike. Undergirding this understanding of the practice are, at least to some extent, data and research. Data collection, beyond allowing for oversight of the practice, plays a critical role in promoting transparency and quality improvement.^7^ Policy‐makers rely on data when crafting policy, as it informs decisions that shape the legal and ethical landscape of end‐of‐life care. By systematically collecting and analyzing demographic, clinical, and procedural data, policy‐makers can derive insights that guide the development, adjustment, and refinement of MAID laws and regulations.

However, scholarly literature,^8^ news articles,^9^ and advocacy press statements^10^ are replete with references to “the data” and what should be extrapolated from it. Advocates and opponents appeal to data as a sine qua non yet often fail to question its validity or relevance to the issue at hand. In one extreme example, opponents of the Canadian MAID regime stake harrowing claims about how the government is effectively euthanizing the poor and how MAID legalization has sparked suicide contagion, but they rely merely on nonvalidated accounts presented in local newspaper articles.^11^ This is not how policy debate should operate. The current debate fails to consider not only the distinction between data and evidence but also whether the data themselves are reliable and to what extent we should be relying on data to guide ethical decision‐making.

As the discourse continues to evolve, a critical analysis of the role of research in shaping these developments becomes imperative. This essay aims to explore the epistemic challenges facing MAID. While the discussion is limited to MAID, the lessons learned are transferable to other bioethical and policy issues. MAID, by virtue of the finality and irreversibility of the procedure, represents a unique and extreme case within bioethics. But this extremity underscores the value it can provide for related issues, such as organ donation and palliative sedation, about which clinicians and others also grapple with irreversibility, mortality, and autonomy.

The essay begins by exploring the current limitations of empirical research on MAID worldwide and the various challenges of conducting research on the subject. I examine the role that researcher and participant bias plays in crafting MAID research, and I also consider the exact extent to which individuals, clinicians, or policy‐makers can and should rely on data when making clinical decisions at the end‐of‐life or when designing MAID policy. I then argue that MAID should be approached with epistemic humility: we should acknowledge our limited ability to comprehend the realities of MAID and that there are limits to what even a high‐quality comprehension of this reality can tell us about how to govern or practice MAID. I close with specific actionable recommendations at the clinical, research, and policy levels to advance the practice of epistemic humility in the age of assisted dying.

## What's Wrong with MAID Data?

Data on MAID comes from two primary sources: state‐sponsored reports and academic scholarship. Governments typically delegate the responsibility of data collection and public reporting to whichever agency is tasked with oversight. This varies across the world, but it typically falls to the public health department, as is the case in Canada, Australia, New Zealand, and the United States, or to an independent agency responsible for MAID oversight, as is the case in the Netherlands and Belgium.^12^ Public reports of MAID activities are typically released annually or biannually, with some jurisdictions doing semiannual reports when first implementing the law. Data collection for oversight typically focuses on patient demographics and on ensuring that safeguards have not been violated.^13^

State‐collected data is limited in a few critical ways. First, the data are observational in nature and are collected retrospectively. These realities limit our ability to manipulate variables to establish causal inference.^14^ For example, state annual MAID reports often include data such as the number of procedures and the reasons for seeking MAID, like unmanageable pain. Observational data may suggest a correlation between high MAID usage and reduced suffering. However, this does not establish cause and effect, as factors like pain‐assessment methods, regional palliative‐care availability, and physician practices could also influence these outcomes. Understanding the causal relationships associated with MAID could provide useful scaffolding for designing better policy and improving practice. Further, lack of causal analysis might lead people to conduct their own causal inference on data that merely describe associations or trends, a phenomenon known as “illusory correlation.”^15^

Second, the data are primarily self‐reported by the attending clinician.^16^ The closest the data come to being validated is the potential triangulation that can be made between data reported by the attending clinician and that collected by a consulting health care worker or participating pharmacist. The Netherlands has a framework that requires attending physicians, upon request, to report to the regional euthanasia review committee either in writing or in person to provide further information about a specific case.^17^ Most cases requiring further testimony are typically deemed “complicated” and are therefore usually included in the final report, including details provided by extra testimony. Otherwise, the data remain unvalidated and thus subject to all the limitations of self‐reported data. Physicians who have violated the law may fraudulently report to oversight agencies, masking their violations. For instance, research into adherence to clinical euthanasia guidelines in the Netherlands has found minor violations go unreported from time to time.^18^

Third, cases may go underreported or unreported. This is not always the result of malice or negligence on the part of the clinician but is instead often due to the realities of end‐of‐life care. In Oregon, data on the duration between ingestion of MAID drugs and death goes unreported in nearly 40 percent of cases, largely because of the absence of a health care worker during MAID. Additionally, there is evidence that cases go unreported in Belgium; one survey found as many as half of all euthanasia cases are not reported to the Federal Control and Evaluation Committee.^19^ There is a gray area between palliative sedation and MAID. In palliative sedation, physicians administer death‐hastening drugs without the intent of hastening death; it is merely a side effect of an effort to relieve pain or suffering. This phenomenon can lead to confusion between the two practices and can affect reporting.^20^

Fourth, there is a lack of transparency in many jurisdictions about how the data are handled.^21^ Most jurisdictions provide the data forms that clinicians, patients, and other MAID participants are required to complete as well as the final reports of that data after oversight has been completed and data has been aggregated. But only a few jurisdictions are clear about how oversight is conducted and what happens to the data between retrieval and reporting. Instead, we are left with an estimate. A more precise interpretation of the state‐reported “total number of MAID deaths” would be “the total number of MAID deaths reported to the state.” Nevertheless, the absence of evidence should not be misconstrued as evidence of absence; skepticism is warranted, yet we should not be dismissive of the possibility that the data accurately reflect the reality.

Academic research has many of the same limitations as do state‐collected data, including being primarily observational and lacking causal analysis. Mara Buchbinder and Cindy Cain highlight four unique contributions that social science research can make to research on MAID: description rather than prescription, understanding cultural meaning, insight into knowledge production, and the ability to compare across jurisdictions for policy insight.^22^ These attributes are invaluable, yet the predominantly qualitative nature of this research limits its usefulness in policy development, particularly for policy regarding the implementation and effectiveness of safeguards in MAID practices. For instance, qualitative interviews of health care providers often explore the perceptions and challenges of implementing safeguards such as ensuring informed consent and assessing decision‐making capacity, but the results of such interviews do not yield data that establish the causal relationship between specific safeguards and patient outcomes across the population. This is in part due to the tendency of qualitative research to focus on theory building and transferability over generalizability, which limits its applicability to policy‐making.^23^ Experimental and quasiexperimental studies on MAID exist, but they are exceptions rather than the rule. In particular, there have been advances in survey sampling that have produced a few experimental studies on MAID.^24^ Cross‐country comparisons have also presented more rigorous investigation into the effects of various policies. In combination, these designs provide a useful template for more research to move toward.

In most cases, empirical MAID research relies on state‐sponsored reports to provide the quantitative components of its data; several recent studies have simply been syntheses of data provided by state‐sponsored agencies.^25^ This research is then subject to the same constraints as the state‐collected data it relies upon. This approach's limited generalizability could pose challenges in forming policies that are both responsive to diverse experiences and grounded in comprehensive evidence.

Research on MAID is particularly susceptible to biases that can influence both the collection and interpretation of data. Researcher bias may impact the framing of study questions, the methodology chosen, and the analysis of results, potentially skewing findings toward preexisting beliefs or hypotheses about MAID.^26^ Studies on controversial topics within MAID, such as rates of suicide in jurisdictions after legalizing MAID, are abound with contradictory results. These discrepancies may be driven by confirmation bias or the authors’ predisposition toward MAID.^27^ Participant biases, including social desirability bias, can lead individuals involved in MAID practices—whether patients, family members, or health care providers—to report experiences in a manner that they perceive as socially acceptable or expected.^28^ Further, the presence of researchers alone can influence patients’ decisions to pursue or not pursue MAID, a phenomenon known as the “Hawthorne effect.”^29^

Researchers, and correspondingly their research, are never unbiased. Although some argue that bias in individual studies is mitigated by the diversity of viewpoints in research literature, thus preventing systemic bias, this assumption does not hold in the context of MAID. Given the limited volume of research on specific MAID‐related questions, the necessary diversity to offset individual biases is lacking. This underscores the necessity for rigorous methodological approaches and critical interpretation of data to ensure the accuracy of research findings.

## The Many‐Headed Beast of MAID Research

Many of these research limitations are not unique to MAID but are common throughout all of social science. However, several unique aspects make research on MAID more difficult than other scientific research, which is already difficult as a baseline. MAID is, by definition, the irreversible. While this trait is shared by various medical procedures, the intended outcome of death elevates the severity of the irreversibility with MAID. This inherently renders MAID patients a unique population and MAID a unique practice. In most cases, patients have a terminal illness, making them a potentially vulnerable population.^30^ Some jurisdictions have allowed or will allow for people without physical ailments, such as individuals with severe treatment‐resistant depression, to receive MAID. These populations warrant more research due to their potential vulnerabilities, especially in the context of informed consent or decision‐making capacity. Research has the potential to add to the emotional and logistical challenges physicians, patients, and family members face in already trying times.

MAID is often set in a highly charged ethical environment. Concerns over privacy and informed consent are amplified due to the sensitive and final nature of MAID.^31^ These issues have led to stringent ethical oversight, potentially limiting the scope of research studies due to the increased risk of harm. State‐sponsored data collection is often constrained by requirements to maximally protect patient privacy.^32^ It is possible that funding agencies and ethics review committees have been skeptical of funding or approving certain types of MAID research in part because of this potential for harm to patients, caregivers, and loved ones.

Recruitment poses its own set of challenges, as potential participants—whether patients, family members, or health care providers—may be reluctant to engage in research on such a personal decision. There is also the potential for a self‐selection bias, with those volunteering to participate in MAID research being among those most favorable to the practice. Legal complexities further compound these challenges, as many researchers may not be aware of the legality of such research or of the practice generally. In the United States, a 1997 ban on the use of federal funds to pay for services that have the purpose of assisting in the death of an individual has muddled the conversation and led many researchers to believe that federal funds cannot be used to support MAID research.^33^ Further, clinicians may be disinclined to participate in research due to these ethical or legal concerns.

Feasibility barriers also pose significant obstacles to MAID research. Evidence‐based law and policy necessitate rigorous studies that generate high‐quality data, with systematic reviews and randomized controlled trials regarded as the studies that produce the most reliable evidence.^34^ Given the low volume of MAID research, the ability to synthesize evidence via literature review or meta‐analysis is limited. Randomizing or manipulating the care provided to the intervention and control groups would require an unethical imposition for patients at the end of life. In addition, there are physical limits to measurement when both the intervention and the outcome are death. Difficulty in attaining adequate sample sizes complicates efforts to draw statistically significant conclusions.

## Data Is Not Destiny

The inherent challenges and limitations of MAID research highlight a fundamental concern over the use of data in policy‐making or ethical decision‐making. The current data we have on MAID may not be an accurate reflection of reality. The truth is that there are inconsistencies and gaps in MAID data and research, even on topics that receive substantial coverage. This is not to dismiss any existing research or current research efforts. In uncertainty, some guidance on which to base decision‐making is better than no guidance, so long as the limitations of that guidance are taken into consideration.

Even if we were to obtain high‐quality evidence that accurately reflects the current state of MAID practice, we would need to remain cautious about its significance and applicability. Scientific evidence, by its nature, can provide insights into the effects and outcomes of MAID but falls short of addressing the ethical complexities inherent in the deeply personal decisions surrounding this practice. As one example, a recent article compared Canada's more liberal MAID regime and the corresponding higher utilization rates with California's stricter regulation and lower utilization, inferring that Canada was “descending rapidly on that slippery slope,” whereas California was “successful.”^35^ The empirical facts (such as utilization trends and the rigidity of safeguards) require ethical assumptions (for instance, that MAID is ethical only when the patient has a terminal illness or that we should protect vulnerable patients from MAID at all costs) in order for one to conclude whether a program is “descending” or is “successful.” The facts of the matter are necessary—but not sufficient in ethical analysis. We must pay attention to the distinction between facts and values and beware of the naturalistic fallacy.^36^ Scientific evidence is not the same as ethical analysis; likewise, conceptions of right and wrong cannot be directly equated with natural properties observable by science.

This is why we should practice epistemic humility in the debate over MAID.

## Embracing Epistemic Humility

Epistemic humility requires four broad cognitive strategies: acknowledging the limits of our knowledge, maintaining suspicion of certainty, practicing reflexivity, and being open to revision and to being wrong. Each of these manifests differently in relation to MAID.

Acknowledging the limits of our knowledge involves recognizing the incomplete understanding across different legal, cultural, and medical frameworks. Our empirical and theoretical grasp of the reality of MAID is limited not only by insufficient scientific knowledge in specific ways but also by the inability of science to determine ethics. The circumstances require us to act on imperfect information. One recent example of policy‐makers practicing epistemic humility is Canada's response to the government‐initiated Expert Panel on MAiD and Mental Illness. Heeding the panel's recommendations for careful assessment, the Canadian parliament delayed until 2027 the provision allowing patients whose sole underlying medical condition is mental illness to access MAID, ensuring time to implement this policy safely and ethically.^37^

Second, we must remain suspicious of certainty when confronting claims about the benefits or harms of MAID. Research findings must be challenged by a rigorous assessment of a study's methods and starting assumptions. This is especially important given the contentious nature of MAID and the potential for research findings to be influenced by political or philosophical positions. All too often, MAID opponents cite data on the effectiveness and availability of palliative care as evidence that MAID is unnecessary, and yet they fail to acknowledge that government reports show that between 75 and 90 percent of MAID patients already receive palliative care. Recently, the governor of Nevada made this very argument in his veto of the state's MAID bill.^38^ If the availability and effectiveness of palliative care is truly the sole reason the governor has for vetoing the bill, then the elected officials who voted for it and the public who supported the law are being robbed based on misconstrued evidence.

Third, researchers and policy‐makers must practice reflexivity by critically examining how their own biases, backgrounds, and societal roles influence their interpretations. In the realm of MAID, where personal values and ethical considerations profoundly shape viewpoints, reflexivity is crucial for mitigating bias and enhancing objectivity. Both clinicians and policy‐makers can demonstrate epistemic humility by being reflexive explicitly—openly acknowledging their positionality in relation to the subject to patients and constituents—or implicitly, through rigorous self‐reflection on the limits of human cognition and the role of emotions in shaping their views on such a sensitive topic.

Fourth, being open to revision and admitting wrongness involves embracing new evidence and alternative viewpoints that may challenge existing beliefs about MAID. This openness is crucial in a field characterized by rapid advancements and changes in public and professional attitudes toward end‐of‐life care.

Integrating these principles of epistemic humility can help to ensure that MAID policies and research are informed by the most reliable evidence available and are responsive to new information and diverse perspectives. This approach not only enhances the legitimacy and acceptability of MAID policies but also respects the complex values and experiences of all stakeholders.

## Clinical, Research, and Policy Recommendations

Positive steps can be taken to put epistemic humility into practice at the clinical, research, and policy levels, but this requires strategic initiatives aimed at improving the depth and integrity of our understanding.

At the clinical level, health care providers who offer MAID to patients should make diligent efforts to stay informed not only on the laws and policies surrounding MAID (as they frequently change) but also on the most recent developments in scientific understanding of the practice. Providers should ensure that they are using the most effective medication regimen. Providers should engage in active reflection about whether their opinion about MAID inappropriately influences their confirmation of various eligibility criteria. Even if a provider believes that particular clinical best practices or laws are harmful, the provider should work to change those recommendations rather than violating them. Organizations like the American Clinicians Academy on Medical Aid‐in‐Dying disseminate educational materials and connect providers through online forums and conferences.^39^

At the research level, there needs to be a more concerted push to expand research efforts beyond attitudinal surveys and into more quasiexperimental and experimental designs. Tools from the social and biomedical sciences can help in creating more generalizable and more actionable research findings that can help inform the ethical and policy deliberations on MAID. The burgeoning subfield of experimental bioethics, in tandem with the subfield of empirical bioethics, has grappled with many issues relevant to building a more robust MAID research infrastructure.^40^ And MAID is not the only subject area plagued by feasibility issues; researchers should look to other areas with similar problems to learn how study design can adapt.

Community‐based participatory research, which uses a collaborative approach to incorporate participants and communities into all aspects of the research, including research design and the impact it will have, offers another route to practice epistemic humility in research.^41^ Innovative research design can address critical concerns such as small sample sizes and the challenge of establishing causality, thus moving researchers closer to an evidence‐based approach to MAID. Lastly, researchers should deliberate about ways that they can bolster transparency and accountability in their research. Several efforts are under way to promote positionality statements or research protocols prior to study implementation. None of these initiatives can function without proper policy in place.

On the policy front, several clear steps can be taken to promote epistemic humility and improve MAID research and practice. Scientific agencies and funders should prioritize academic research into MAID as well as its role in the large spectrum of end‐of‐life care options, especially as populations age. Beyond traditional funding mechanisms, governments should aim to establish more international collaboration on the topic through consortia, conferences, and data repositories. International collaboration would allow for better comparison of different regulations as well as allow greater statistical power for investigations into shared characteristics or causal mechanisms. Governments also need to clarify their laws on MAID research, and promoting the institutionalization of research can help streamline efforts and ensure that studies are conducted ethically and effectively.^42^ Investment should be made in better state‐sponsored data collection to capture the complexity of MAID practices and outcomes accurately.^43^ This will both enhance oversight and provide higher quality data for academic research.

Having a policy ecosystem that is responsive to evidence and ethics requires both accessible and valid research as well as mechanisms for policies to be reviewed. An example of the latter is the inclusion of a sunset clause in the California MAID legislation. Although there are drawbacks to having an expiration date on legislation, it serves to ensure that policy‐makers are adaptive to changing circumstances.^44^ Alternatively, there are less extreme options, such as mandating that independent committees meet to review the law and propose recommendations on a set timeline.

## Epistemic Humility as an Antidote

Currently, the evidence base for MAID is not as robust as it could be. Concerted efforts by policy‐makers, health care professionals, and academics across medicine, public health, philosophy, and public policy are needed to strengthen the foundation upon which MAID policies and practices are built. Striving for evidence‐based MAID means not only improving the quantity and quality of research but also making a cultural shift in how we approach research and use data. Few topics are as polarizing as MAID; and bias, manipulated data, and bad science poison the debate. This all suggests the importance of adopting a broader perspective that incorporates, but is not limited to, empirical evidence in guiding MAID policies and practices.

Epistemic humility is an antidote to such challenges. Meeting them requires accepting that complexity is an inherent characteristic of MAID. This requires learning how to make decisions when faced with uncertainty. This requires the bravery to admit that we do not know everything. Such an approach can do more than mitigate these challenges; it can transform them into opportunities for more ethical, informed, and compassionate engagement with the issue of MAID. This is what it means to practice epistemic humility in the age of assisted dying.

## Acknowledgments

I am grateful to reviewers of this journal and to the International Conference on End‐of‐Life: Law, Ethics, Policy, and Practice community for feedback on the paper.
